# Could electrodermal activity detect emotions soon?

**DOI:** 10.2478/joeb-2025-0001

**Published:** 2025-01-15

**Authors:** Haval Y. Y. Aldosky, Dindar S. Bari

**Affiliations:** Department of Physics, College of Science, University of Duhok, Duhok, Kurdistan region, Iraq; Scientific Research Center, University of Zakho, Zakho, Kurdistan region, Iraq; Department of Physics, College of Science, University of Zakho, Zakho, Kurdistan region, Iraq

Electrodermal activity (EDA) refers to the changes in active and passive electrical properties across the skin in areas of the body that are psychologically responsive [1]. Studies of the EDA date back more than 100 years ago when researchers first identified a link between the flow of current in the skin and psychological states. However, the essential discovery of EDA phenomena is credited to two researchers the French neurologist Féré (1888) and the Russian physiologist Tarchanoff (1889), who likely worked without knowledge of each other. Féré, using an external direct current, observed a reduction in skin resistance following emotional stimulation. On the other hand, Tarchanoff, without utilizing any external source of current found changes in skin potential as a result of emotional stimulation [2]. The first connection between EDA and emotions can be associated with the discoveries of Féré and Tarchanoff. However, Jung and his colleagues (1906) in the first scientific study used EDA to evaluate patients' emotional sensitivity during word association tasks [3]. These early works laid the foundation for modern psychophysiology and by the early 1970s several studies had been performed on using EDA for detecting emotional states. Today EDA can be considered one of the popular approaches for investigating human psychophysiological emotion.

EDA plays a significant role in psychophysiological measurements and is applied in various clinical applications. EDA measurements are considered an invaluable tool for assessing changes in autonomic reactivity, especially the sympathetic branch evoked by different psychosocial events such as stress, emotion, attention, anxiety, etc. Two key features enhance the usefulness of EDA in psycho-physiological applications. Firstly, EDA signals reflect only the sympathetic activity of the autonomous nervous system and there is no parasympathetic innervation of sweat glands. Secondly, neurotransmission at the effector synapse is predominantly cholinergic, meaning it is mediated by acetylcholine release [4].

EDA measurements are considered to be effective in investigating stress and anxiety compared to other physiological measurements like heart rate, respiration rate, and skin temperature [5]. Studies have shown that changes in EDA responses are known to be associated with emotional arousal [6, 7]. Their ability to provide real-time data in a non-invasive way led to applications in stress detection [8,9,10], autism examination [11], detection of depression [12], schizophrenia prognosis [13], recognizing emotional states [14], seizure detection [15], etc.

Advancements in EDA recording technologies and the creation of innovative, reliable signal processing algorithms have sparked a growing interest in using EDA measurements across various new and emerging fields. More recent uses of EDA measurements in psychophysiological applications include continuous (long-term evaluation) stress monitoring [16], automated mental workload monitoring [17], sleep drowsiness and driver safety [18], and measures of sympathetic nervous system activity [19].

Based on the aforementioned, it can be said that EDA is rapid, inexpensive, non-invasive, and offers unbiased information on sympathetic nervous system arousal [20]. EDA has made great strides in psychophysiological assessments, although emotional measurements still present certain challenges. Most of these challenges contribute to inaccurate emotional behavior determination, even though EDA monitors sympathetic nervous system arousal and is frequently employed as an indirect indicator of emotional states. Some of these limitations are explained as follows:

EDA measures physiological arousal, which is linked to the activation of the sympathetic nervous system. However, as with Multiple Arousal Theory [21], it cannot accurately distinguish between different types of emotions (e.g., anger, fear, happiness, neutral, sad, etc.) as they may produce similar arousal patterns [22]. In other words, EDA requires complementary data sources to assess the emotion appropriately, which makes it challenging to recognize the emotional valence (positive or negative) without further measurements.

The non-emotional factors are another limitation; EDA can be impacted by factors unrelated to emotions, such as physical activity or movement [23], ambient temperature [24] or humidity [25], sweat gland density, and caffeine intake [23].

Individual differences in physiology and baseline EDA levels are another significant factor that contributes to response variability. These factors include age, gender [26, 27], and mental health conditions (e.g., depression or anxiety disorders) [28].

Technical challenges also affect the efficacy of EDA in psychological research and its applications. Sensitivity to electrode placement [29], skin contact [30], and motion artifacts are among these technical challenges. The low satisfaction of users and reliability, particularly in wearable or portable applications, necessitates improvements in signal processing and sensor design development.

EDA devices are expected to be developed to detect emotional states more accurately soon, assuming that these challenges have been addressed and integrated with multimodal systems like emotion-focused AI models and in combination with other physiological signals like EEG, ECG, heart rate variability, respiration (RSP), skin temperature, photoplethysmography, and eye tracking (ET). Also, concurrently monitoring EDA laterality would aid in gaining a deeper comprehension [21]. This will bring them one step closer to devices that can sense an individual's feelings and potentially lead to a deeper understanding of human emotions [31]. While EDA measurements of real-time emotional feedback are making their forays into personalized medicine and even brain function, the regulatory framework must advance similarly for applications that are safe, effective, and ethically responsible.

In the future, EDA measurements are expected to be crucial in the following areas: detecting emotions, and monitoring physiological signals continuously to identify and track mental health conditions like stress, anxiety, or depression; EDA-enabled wearables could soon enhance a deeper understanding of human behavior and brain function; improving educational experiences by adjusting to students' emotional states during learning; and enhancing communication and collaboration with robots, making them more perceptive and empathetic.

## References

[1] Boucsein W (2012). Electrodermal activity.

[2] Neumann E, Blanton R (1970). The early history of electrodermal research. Psychophysiology.

[3] Dawson ME, Schell AM, Filion DL, Cacioppo JT, Tassinary LG, Berntson GG (2007). The electrodermal system. Handbook of Psychophysiology.

[4] Gellman MD (2020). Encyclopedia of behavioral medicine.

[5] Tronstad C, Amini M, Bach DR, Martinsen ØG (2022). Current trends and opportunities in the methodology of electrodermal activity measurement. Physiol Meas.

[6] Li S, Sung B, Lin Y, Mitas O (2022). Electrodermal activity measure: A methodological review. Ann Tour Res.

[7] Bari DS, Rammoo MN, Aldosky HY, Jaqsi MK, Martinsen ØG (2023). The five basic human senses evoke electrodermal activity. Sensors.

[8] Stržinar Ž, Sanchis A, Ledezma A, Sipele O, Pregelj B, Škrjanc I (2023). Stress detection using frequency spectrum analysis of wrist-measured electrodermal activity. Sensors.

[9] Bari DS, Aldosky HY, Tronstad C, Martinsen ØG (2021). The correlations among the skin conductance features responding to physiological stress stimuli. Skin Res Technol.

[10] Bari DS, Rammoo MN, Youssif AA, Najman HM, Aldosky HY, Tronstad C (2024). Electrodermal Activity for Quantitative Assessment of Dental Anxiety. J Sens Actuator Netw.

[11] Ferguson BJ, Hamlin T, Lantz JF, Villavicencio T, Coles J, Beversdorf DQ (2019). Examining the association between electrodermal activity and problem behavior in severe autism spectrum disorder: A feasibility study. Front Psychiatry.

[12] Kim AY, Jang EH, Kim S, Choi KW, Jeon HJ, Yu HY (2018). Automatic detection of major depressive disorder using electrodermal activity. Sci Rep.

[13] Schell AM, Dawson ME, Rissling A, Ventura J, Subotnik KL, Gitlin MJ (2005). Electrodermal predictors of functional outcome and negative symptoms in schizophrenia. Psychophysiology.

[14] Al Machot F, Elmachot A, Ali M, Al Machot E, Kyamakya K (2019). A deep-learning model for subject-independent human emotion recognition using electrodermal activity sensors. Sensors.

[15] Vieluf S, Amengual-Gual M, Zhang B, El Atrache R, Ufongene C, Jackson MC (2021). Twenty-four-hour patterns in electrodermal activity recordings of patients with and without epileptic seizures. Epilepsia.

[16] Meijer AL, Arts LP, Gomez R, van den Broek EL (2023). Electrodermal activity: A continuous monitor of well-being. J Smart Cities Soc.

[17] Ghaderyan P, Mirzaeian R (2024). New oscillatory features of electrodermal activity signals evaluated in automated mental workload monitoring. Biomed Signal Process Control.

[18] Malathi D, Jayaseeli JD, Madhuri S, Senthilkumar K (2018). Electrodermal activity based wearable device for drowsy drivers. J Phys Conf Ser.

[19] Van Der Mee DJ, Gevonden MJ, Westerink JH, De Geus EJ (2021). Validity of electrodermal activity-based measures of sympathetic nervous system activity from a wrist-worn device. Int J Psychophysiol.

[20] Matamala A, Soler-Vilageliu O, Iturregui-Gallardo G, Jankowska A, Méndez-Ulrich JL, Serrano A (2020). Electrodermal activity as a measure of emotions in media accessibility research: methodological considerations. J Spec Transl.

[21] Bjørhei A, Pedersen FT, Muhammad S, Tronstad C, Kalvøy H, Wojniusz S (2019). An investigation on bilateral asymmetry in electrodermal activity. Front Behav Neurosci.

[22] Rahman JS, Hossain MZ, Gedeon T Measuring Observers' EDA Responses to Emotional Videos.

[23] Hossain M-B, Kong Y, Posada-Quintero HF, Chon KH, Edlund JE, Nichols AL (2024). Electrodermal Activity: Applications and Challenges. The Cambridge Handbook of Research Methods and Statistics for the Social and Behavioral Sciences.

[24] Qasim MS, Bari DS, Martinsen ØG (2022). Influence of ambient temperature on tonic and phasic electrodermal activity components. Physiol Meas.

[25] Bari DS, Aldosky HY, Tronstad C, Kalvøy H, Martinsen ØG (2018). Influence of relative humidity on electrodermal levels and responses. Skin Pharmacol Physiol.

[26] Bari DS (2020). Gender differences in tonic and phasic electrodermal activity components. Sci J Univ Zakho.

[27] Aldosky HY (2019). Impact of obesity and gender differences on electrodermal activities. Gen Physiol Biophys.

[28] Pruneti C, Fiduccia A, Guidotti S (2023). Electrodermal activity moderates the relationship between depression and suicidal ideation in a group of patients with anxiety and depressive symptoms. J Affect Disord.

[29] Braithwaite JJ, Watson DG, Jones R, Rowe M (2013). A guide for analysing electrodermal activity (EDA) & skin conductance responses (SCRs) for psychological experiments. Psychophysiology.

[30] Hossain MB, Kong Y, Posada-Quintero HF, Chon KH (2022). Comparison of electrodermal activity from multiple body locations based on standard EDA indices’ quality and robustness against motion artifact. Sensors.

[31] Vo DK, Trinh KT (2024). Advances in Wearable Biosensors for Healthcare: Current Trends, Applications, and Future Perspectives. Biosensors.

